# Traumatic Brain Injury in Aged Mice Induces Chronic Microglia Activation, Synapse Loss, and Complement-Dependent Memory Deficits

**DOI:** 10.3390/ijms19123753

**Published:** 2018-11-26

**Authors:** Karen Krukowski, Austin Chou, Xi Feng, Brice Tiret, Maria-Serena Paladini, Lara-Kirstie Riparip, Myriam M. Chaumeil, Cynthia Lemere, Susanna Rosi

**Affiliations:** 1 Department of Physical Therapy and Rehabilitation Science, University of California, San Francisco, CA 94143, USA; karen.krukowski@ucsf.edu (K.K.); austinc06@gmail.com (A.C.); xi.feng@ucsf.edu (X.F.); brice.tiret@ucsf.edu (B.T.); paladini.mariaserena@gmail.com (M.-S.P.); lriparip@gmail.com (L.-K.R.); myriam.chaumeil@ucsf.edu (M.M.C.); 2 Brain and Spinal Injury Center, University of California, San Francisco, CA 94110, USA; 3 Department of Radiology and Biomedical Imaging, University of California San Francisco, Surbeck Laboratory of Advanced Imaging, San Francisco, CA 94143, USA; 4 Ann Romney Center for Neurologic Diseases, Brigham & Women’s Hospital, Boston, MA 02115, USA; clemere@bwh.harvard.edu; 5 Harvard Medical School, Boston, MA 02115, USA; 6 Department of Neurological Surgery, University of California, San Francisco, CA 94110, USA; 7 Weill Institute for Neuroscience, University of California, San Francisco, CA 94143, USA; 8 Kavli Institute of Fundamental Neuroscience, University of California, San Francisco, CA 94143, USA

**Keywords:** traumatic brain injury, complement, C1q, microglia, synapse

## Abstract

Traumatic brain injury (TBI) is of particular concern for the aging community since there is both increased incidence of TBI and decreased functional recovery in this population. In addition, TBI is the strongest environmental risk factor for development of Alzheimer’s disease and other dementia-related neurodegenerative disorders. Critical changes that affect cognition take place over time following the initial insult. Our previous work identified immune system activation as a key contributor to cognitive deficits observed in aged animals. Using a focal contusion model in the current study, we demonstrate a brain lesion and cavitation formation, as well as prolonged blood–brain barrier breakdown. These changes were associated with a prolonged inflammatory response, characterized by increased microglial cell number and phagocytic activity 30 days post injury, corresponding to significant memory deficits. We next aimed to identify the injury-induced cellular and molecular changes that lead to chronic cognitive deficits in aged animals, and measured increases in complement initiation components C1q, C3, and CR3, which are known to regulate microglial–synapse interactions. Specifically, we found significant accumulation of C1q on synapses within the hippocampus, which was paralleled by synapse loss 30 days post injury. We used genetic and pharmacological approaches to determine the mechanistic role of complement initiation on cognitive loss in aging animals after TBI. Notably, both genetic and pharmacological blockade of the complement pathway prevented memory deficits in aged injured animals. Thus, therapeutically targeting early components of the complement cascade represents a significant avenue for possible clinical intervention following TBI in the aging population.

## 1. Introduction

Traumatic brain injury (TBI) affects 3–5 million individuals each year in the United States alone. TBI is one of the most powerful environmental risk factors associated with the development of dementia. Elderly individuals are particularly vulnerable to traumatic brain injury, and the aging brain is more susceptible to chronic inflammatory/degenerative changes following TBI. As the aging population continues growing, the burden of TBI in the elderly is expected to increase dramatically. The leading causes of TBI include falls in everyday activity, followed by motor vehicle accidents, and the prevalence and severity of TBIs increases within the aging population [1,2,3]. The deleterious outcomes from TBI include loss of motor function, cognitive decline, reduced quality of life, and in severe cases death, all of which worsen with age [4,5,6].

It was demonstrated both in rodents and humans that, after injury, there is a robust inflammatory signature in the brain [7,8,9,10,11,12] that is significantly more pronounced and persistent [7,9] in the aged brain. During initial screening for specific inflammatory signatures, C1q, the initiating molecule of the classical complement cascade, was found to be significantly modulated by trauma in the aging brain [9]. C1q was shown to localize on synapses and work as a signaling molecule to induce synapse elimination by microglial cells [13,14]. Recent reports linked aberrant C1q overexpression and consequent synapse loss in models of neurodegenerative disease including Alzheimer’s, and a viral infection model [15,16,17,18,19,20]. Notably, C1q expression increases with age throughout the brain [21]; however, the possible role that chronic complement initiation and the consequent microglia response play in the aged injured brain remains unknown.

Despite the established demographic of TBI in the aging population and the increase in life expectancy, there remains a paucity of studies investigating the mechanisms that lead to worse outcome. Due to the persistent and exacerbated inflammatory response in the aged brain after injury [7,9], it is imperative to investigate the possible mechanisms in order to find therapeutic targets for intervention. Here, we utilize a focal contusion injury model to identify the mechanistic relationships between microglia activation, complement initiation, synapse loss, and cognitive decline in aged injured animals.

## 2. Results

### 2.1. Lesion Progression after Contusion Injury in Aged Animals

**Figure 1:**
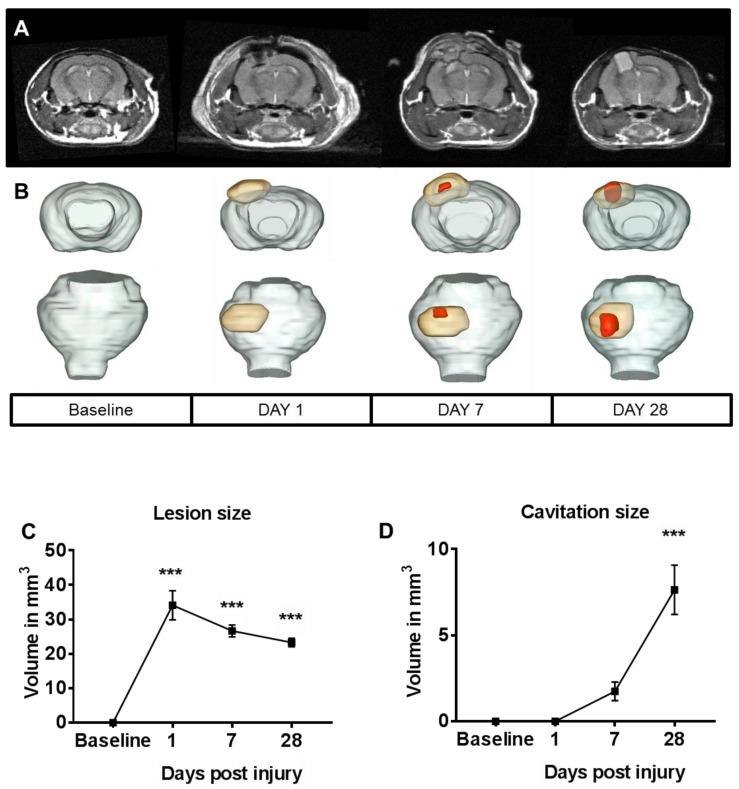
T_2_-weighted magnetic resonance imaging (MRI) monitors longitudinal changes in lesion size and cavitation after contusion injury in aged animals. (**A**) Axial T_2_-weighted magnetic resonance (MR) representative images of an injured animal, acquired a day prior to injury (baseline), and on days 1, 7, and 28 post injury on a 14.1-tesla MRI system using the following parameters: echo time (TE)/repetition time (TR) = 12/2000 ms; slice thickness = 0.5 mm; number of averages = 8; matrix = 256 × 256; field of view (FOV) = 30 × 30 mm^2^; acquisition time = 8 min. (**B**) Corresponding three-dimensional (3D) rendering of the lesion (orange) and cavitation (red) over time for the same injured animal. (**C**) Lesion size (expressed in mm^3^, defined as a mix of hyper- and hypo-intense contrasts) was measured starting at one day post injury. One-way ANOVA revealed significant differences (F = 47.77; *p* < 0.0001). Tukey post hoc test revealed differences between groups; *n* = 5–6/time point. (**D**) Cavitation (expressed in mm^3^, defined as hyperintense contrasts) was measured starting at seven days, and peaked at 28 days post injury. One-way ANOVA was used for non-parametric analysis (Kruskal–Wallis test), and Dunn’s multiple comparison test was used post hoc. Error bars represent group means and standard errors of the mean (SEM); *** *p* < 0.01.

**Figure 2:**
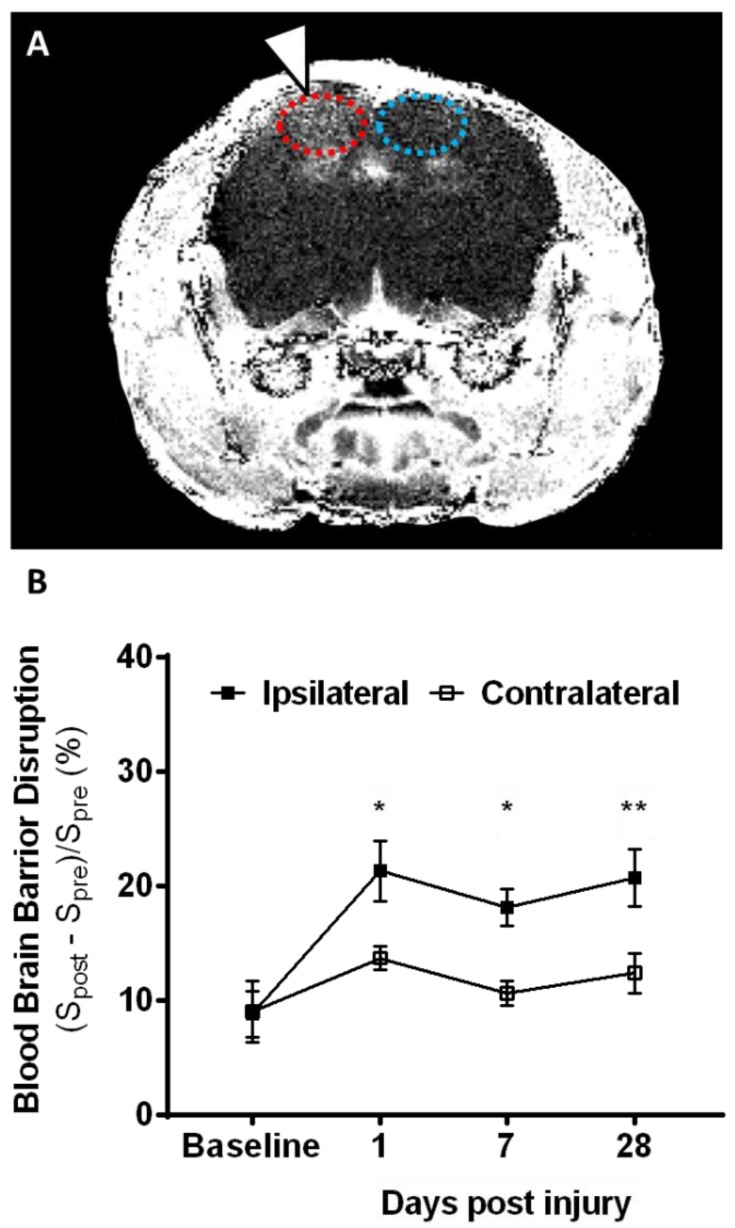
Post-contrast T_1_-weighted MRI detects persistent blood–brain barrier (BBB) damage after contusion injury in aged animals. (**A**) Enhancement ratio map acquired at 28 days post injury (white arrow), as calculated from T_1_-weighted magnetic resonance (MR) images acquired pre- and post-injection of 1 mmol/kg Magnevist^®^ on a 14.1-tesla MRI system using the corresponding parameters: TE/TR = 4.6/112 ms; slice thickness = 0.5 mm; number of averages = 30; flip angle = 10; matrix = 256 × 192; field of view (FOV) = 25 × 25 mm^2^; acquisition time = 10 min 41 s. Regions of interest (ROIs) used for quantification of ipsilateral (red) and contralateral (blue) signals are superimposed. (**B**) Quantification of the mean intensity ratio, where *S_pre_* and *S_post_* represent the signal intensity before and after contrast injection, in ipsilateral (closed black squares) and contralateral (open black squares) ROIs shows a significant increase in enhancement in the ipsilateral ROI at every time point post injury. Error bars represent means and SEM. Two-way repeated measure ANOVA found significant differences in the time effect (F = 4.575, *p* < 0.05) and brain region effect (F = 32.24, *p* < 0.0001), as well as their interaction (F = 3.77, *p* < 0.05), with Sidak correction. * *p* < 0.05; ** *p* < 0.01.

Mild to moderate focal contusion injury to 19-month-old animals was examined with the highly reproducible controlled cortical injury (CCI) model [7,8,9,22,23]. Using a combination of T_2_-weighted and pre/post-contrast T_1_-weighted magnetic resonance imaging (MRI) methods, where T1 and T2 are the longitudinal and transverse relaxation time respectively, we investigated lesion size, cavitation, and blood–brain barrier (BBB) breakdown over time (Figure 1 and Figure 2). On T_2_-weighted MRI, lesions appear as a mix of hyper- and hypo-intense contrasts, delineating a measured total lesion size. On the same images, well-delineated hyperintense cavitation can be seen on the injured hemisphere. Increased lesion size and cavitation measured by one day post injury (dpi) persisted for up to 28 dpi. While lesion size peaked at one dpi and then plateaued by 28 dpi, cavitation volume kept growing and, by one month, reached a volume of ~8 mm^3^ (Figure 1A,B). Next, we measured BBB breakdown following focal contusion injury using T_1_-weighted MRI post injection of gadolinium-based contrast agent, as commonly used in the clinical setting. In the aged brain, BBB integrity was compromised after injury, as shown by increased accumulation of contrast agent through vascular leakage. BBB breakdown was measured for up to one month post injury (Figure 2). Overall, as expected, lesion size and cavitation were larger in aged animals than that published previously for younger animals [23].

### 2.2. Microglia Numbers Increase Chronically after Contusion Injury

**Figure 3:**
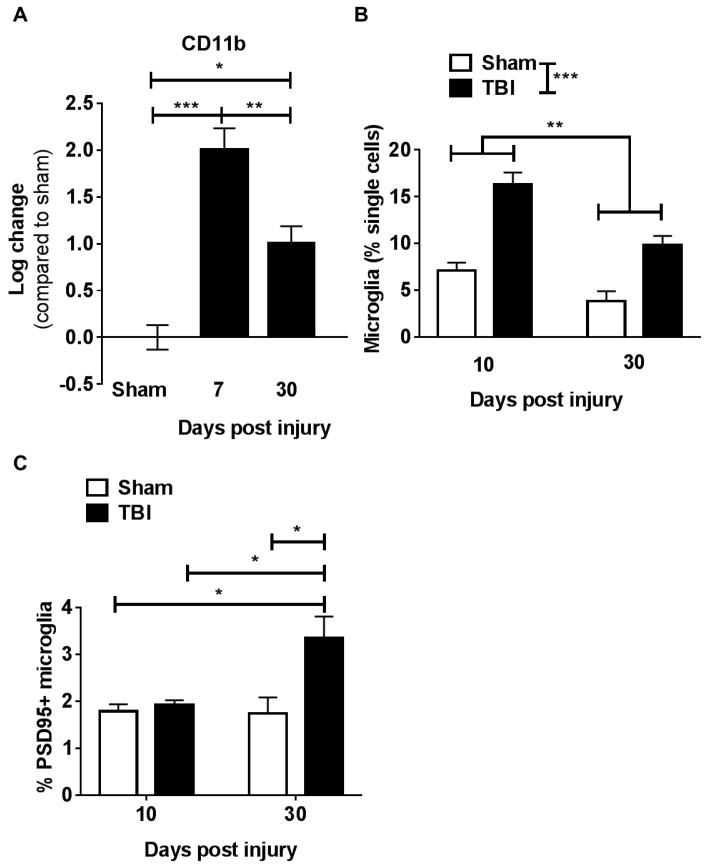
Increased microglia numbers and phagocytic activity chronically after contusion injury. (**A**) Cluster of differentiation 11b (CD11b) gene-expression changes in the hippocampus of aged animals were measured by qPCR analysis, comparing expression levels between sham uninjured animals, and those 7 and 30 days post injury. CD11b, a marker for microglia and macrophages, significantly increases post injury. One-way ANOVA revealed significant differences (F = 21.12; *p* < 0.0001). Tukey post hoc test revealed differences between groups; *n* = 4–6/group (**B**) Brain microglia composition increases at 10 and 30 days post injury. Flow cytometry result revealed that percentage of microglia in the brain significantly increased at both 10 and 30 days after traumatic brain injury (TBI). Two-way ANOVA revealed significant effects of time (F(1,8) = 21.93, *p* < 0.01) and injury (F(1,8) = 53.59, *p* < 0.0001) without significant interaction (F(1,8) = 2.341, *p* = 0.1645). (**C**) Changes in synaptosomes (containing post-synaptic density protein PSD95) phagocytosis by microglia. There was no change in microglia phagocytosis activity at 10 days after TBI. However, at 30 days after TBI, there was a significant increase of exogenous synaptosome phagocytosis activity in microglia. Two-way ANOVA revealed significant differences in the effects of time (F = 5.333, *p* < 0.05) and injury (F = 8.456, *p* < 0.05), with significant interaction (F = 6.111, *p* < 0.05). Bars depict group means and SEM; * *p* < 0.05, ** *p* < 0.01, *** *p* < 0.0001.

We and others previously reported that, in the injured brain of aging animals, there is an increase in microglia when compared to brains from young injured animals (three months of age) for up to seven days following TBI [7,10]. Here, we used two independent methods, measuring messenger RNA (mRNA) with quantitative PCR (qPCR) and protein levels with flow cytometric staining, to determine whether the increase in microglia observed at 7 dpi persists at later time points when cognitive deficits are also observed [7]. We measured an increase in cluster of differentiation 11b (CD11b, which together with CD18 forms the complement receptor 3 or CR3) mRNA expression in the injured hippocampus beginning at 7 dpi and lasting up to 30 days after injury when compared to aged-matched sham animals (Figure 3A). These results were further confirmed using flow cytometric staining for microglia (CD45^low^, CD11b^+^). We quantified a significant increase in microglial numbers compared to aged sham animals both at 10 and 30 dpi (Figure 3B).

### 2.3. Contusion Injury Exacerbates Microglia Phagocytic Activity in the Aged Brain

We next investigated the phagocytic properties of microglia in the aged brain by a newly modified in vivo engulfment assay [24]. In this assay, fluorescent antibody-labeled post-synaptic density protein 95 (PSD-95) synapse particles were injected into the injured hippocampus of the aged sham or injured animals. Three days after injection, microglia were isolated and phagocytic activity quantified by measuring the percentage of microglia that engulfed labeled synapses (CD45^low^, CD11b^+^ co-expressing with intracellular PSD-95/fluorescein isothiocyanate (FITC)). We found a significant increase in the percentage of microglia that engulfed labeled synapses in aged injured animals at 30 dpi when compared with age-matched sham controls (Figure 3C). Importantly, the increase in microglia-engulfing activity was limited to chronic endpoints, as we did not detect differences between sham and TBI animals at 10 dpi. These results suggest that the injury-induced changes in the aged brain take place progressively after injury and could be responsible for more severe long-term cognitive outcomes.

### 2.4. Contusion Injury Induces Robust Complement Initiation in the Aged Brain

**Figure 4:**
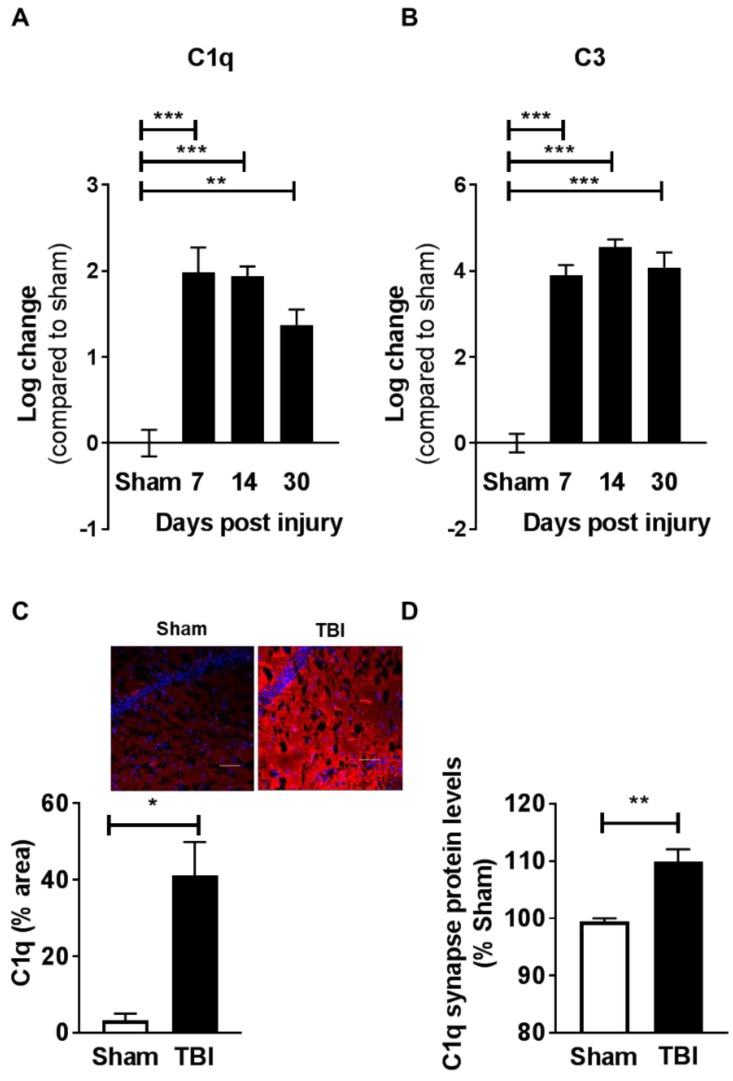
Contusion injury induces robust complement initiation in the aged brain. Complement initiation components (**A**) C1q and (**B**) C3 gene-expression changes in the hippocampus of aged animals were measured by qPCR analysis, comparing expression levels between sham animals, and those 17, 14, and 30 days post injury. Both C1q and C3 expression levels were increased after injury. One-way ANOVA revealed significant differences (C1q-F = 15.89; *p* < 0.0001; C3-F = 50.58; *p* < 0.0001). Bonferroni post hoc test revealed differences between groups; *n* = 4–6/group. (**C**) C1q protein expression was measured by immunohistochemical staining in the dorsal hippocampus. Representative images from sham and TBI (30 dpi) animals (inset, in red C1q staining and in blue cell body counterstaining). C1q protein expression increased chronically after injury. No reactivity was observed in secondary alone. White scale bar = 50 µm; 200× magnification; *n* = 4–6/group. (**D**) Hippocampi were collected and synaptosomes were isolated by sucrose gradient followed by size calibration beads. C1q co-localization of synapse was measured by antibody staining. TBI (30 dpi) significantly increases C1q accumulation at synapse when compared with sham animals. Student’s *t*-test with Welch’s correction was used to measure differences between groups; *n* = 6/group. Bars depict group means and SEM; * *p* < 0.05, ** *p* < 0.01, *** *p* < 0.01.

To follow up on recent reports linking microglia and complement initiation in different neurodegenerative conditions [15,16,17], we next investigated the effect of injury on C1q expression over time. Using qPCR analysis, we measured significant increases in C1q mRNA expression and a downstream complement factor C3 within the injured hippocampus, beginning at seven days and up to 30 days after injury (Figure 4A,B). The chronic induction of C1q was further confirmed by immunohistochemistry analysis of the hippocampus (CA1) (Figure 4C). Notably, using flow synaptocytometry we demonstrate that individual synapses co-localized with elevated protein levels of C1q at 30 dpi (Figure 4D). Furthermore, complement initiation was contained to the injured, ipsilateral hippocampus, as only small increases in C1q and no increase in C3 were measured at 30 dpi in the contralateral hemisphere when compared with the sham animals (Figure S1).

### 2.5. Contusive Injury Results in Chronic Synapse Loss in the Aged Brain

**Figure 5:**
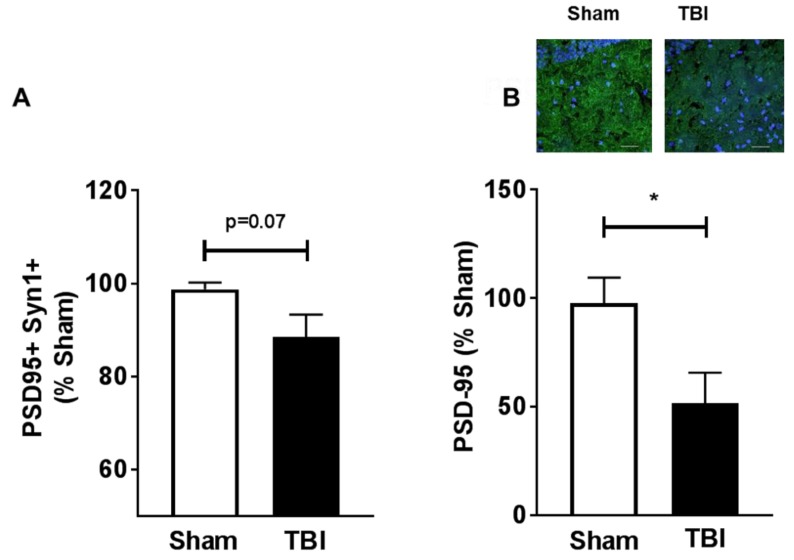
Contusive injury results in chronic synapse loss in the aged brain. (**A**) Hippocampi were collected and synaptosomes were isolated by sucrose gradient followed by size calibration beads, before the co-expression of pre- and post-synaptic markers. The pre-synaptic marker was Synapsin-1 (Syn-1) and the post synaptic marker was post-synaptic density protein 95 (PSD-95). Significant decreases were found in total synaptosome numbers in the TBI (30 dpi) group when compared to sham group; *n* = 8–9/group. (**B**) Synaptic levels were measured by PSD-95 staining in the dorsal hippocampus. Representative images are shown in the inset. Aged TBI (30 dpi) animals had reduced PSD-95 expression. No reactivity was observed in secondary alone. White scale bar = 30 µm; 200× magnification; *i* = 4/group (PSD95 in green and cell bodies in blue). Student’s *t*-test was used to measure differences. Bars depict group means and SEM; * *p* < 0.05.

C1q localization at the synapse was shown to be directly associated with decreases in synapse numbers [16]; therefore, we next investigated if chronic C1q accumulation (30 dpi) was associated with hippocampal synapse loss after injury in aged animals. Synapse numbers in the hippocampus were assessed by flow cytometric staining [25,26,27] after synapse isolation. Synapsin-1 was used as a standard pre-synaptic marker, and PSD-95 was used as a post-synaptic marker. We measured a strong trend for reduced synapse numbers in the aged injured animals when compared to the age-matched sham animals (Figure 5A). The decreases, measured by cytometric analysis, were further confirmed by immunohistochemistry staining for the post-synaptic marker PSD-95 (Figure 5B).

### 2.6. Complement Blockade Prevents Memory Deficits in Aged Animals after Contusion Injury

**Figure 6:**
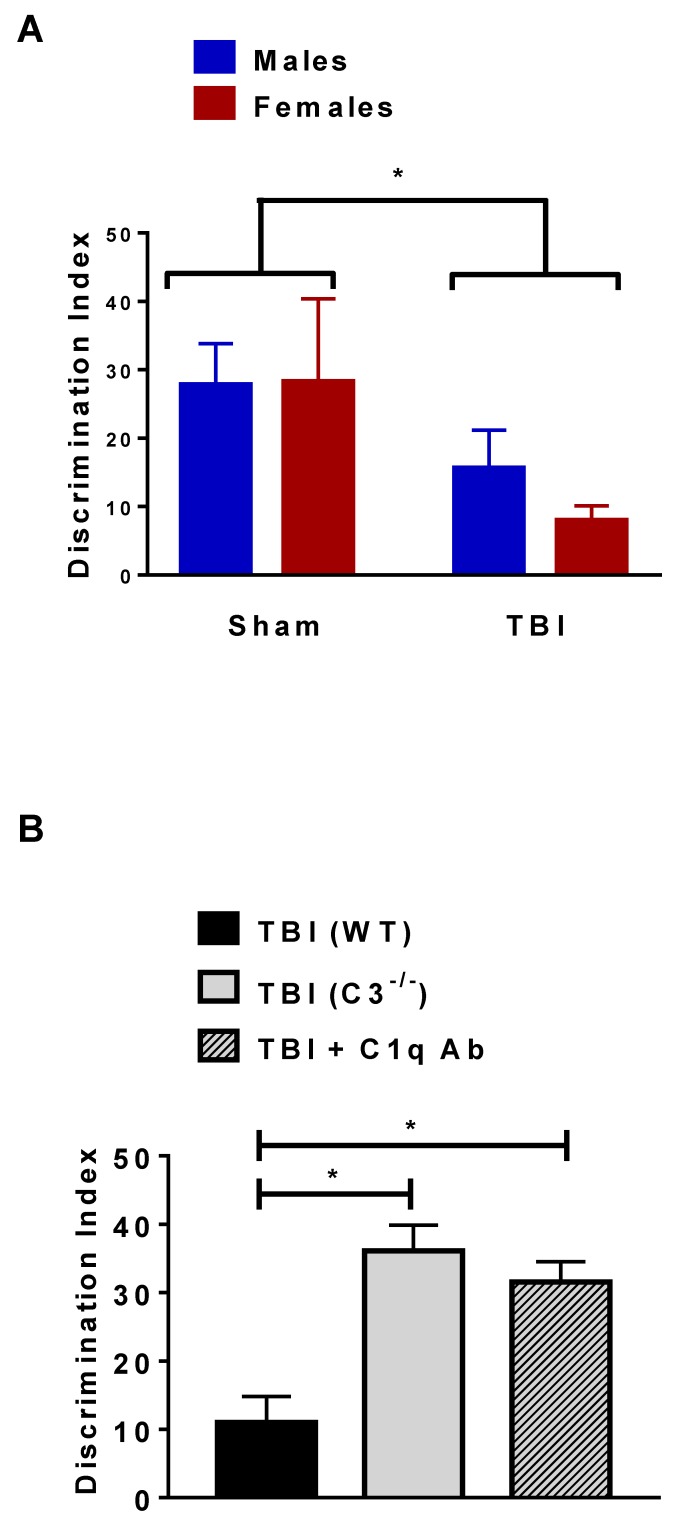
Complement blockade prevents memory deficits in aged animals after contusion injury. Trauma-induced memory deficits were measured by novel object recognition. Animals were exposed to two identical objects; 24 h later, the animals were exposed to one familiar object and one novel object. Memory deficits were calculated by a deficit in distinguishing the new (novel) object. (**A**) Both male and female animals displayed memory deficits following TBI (30 dpi). Two-way ANOVA revealed a significant injury effect (F = 4.42, *p* < 0.05); *n* (males) = 15–17/group; *n* (females) = 6–7/group. (**B**) Genetic C3 knock-out (C3^−/−^) or pharmacological (C1q-inhibiting antibody (Ab)) blockade of complement initiation factors prevents trauma-induced deficits; *n* (TBI wild-type) = 27 mice; *n* (TBI C3^−/−^) = 4 mice; *n* (TBI + C1q Ab) = 7 mice. One-way ANOVA revealed significant differences (F = 6.35; *p* < 0.01). Tukey post hoc analysis was used for differences between groups. * *p* < 0.05.

To determine the potential role that increased complement expression plays in the development of injury-induced chronic memory deficits, we used two complementary approaches: (1) a genetic approach, with aged C3 knock-out (C3^−/−^) mice, and (2) pharmacological intervention with an anti-C1q antibody [16]. Recognition memory at 30 dpi was measured using the novel object recognition test. This task does not have aversive stimuli and it is based on the innate tendency of the rodents to explore novelty. After being habituated to be in an open arena, animals were allowed to explore two identical objects for 10 min; 24 h later, they were exposed to one of the previously encountered objects (familiar) and to a novel one. Recognition memory was determined by a preference for time spent exploring the novel object compared to the familiar one. Aged, injured animals were significantly impaired in their ability to recognize the novel versus the familiar object compared to sham-matched animals, denoting memory impairments (Figure 6A). Importantly, we observed memory deficits in both male and female aged, injured animals and no sex-dependent differences (Figure 6A). Notably, C3^−/−^ aged injured animals and aged wild-type injured animals that received the C1q blocking antibody (administered weekly) displayed significantly improved recognition memory capabilities when compared with the aged, injured wild-type animals (Figure 6B). These data demonstrate that activation of the complement initiation pathway plays a significant role in the development of memory deficits in the injured aged animals. Most importantly targeting the complement initiation pathway can prevent chronic trauma-induced deficits in aged animals.

## 3. Discussion

**Figure 7:**
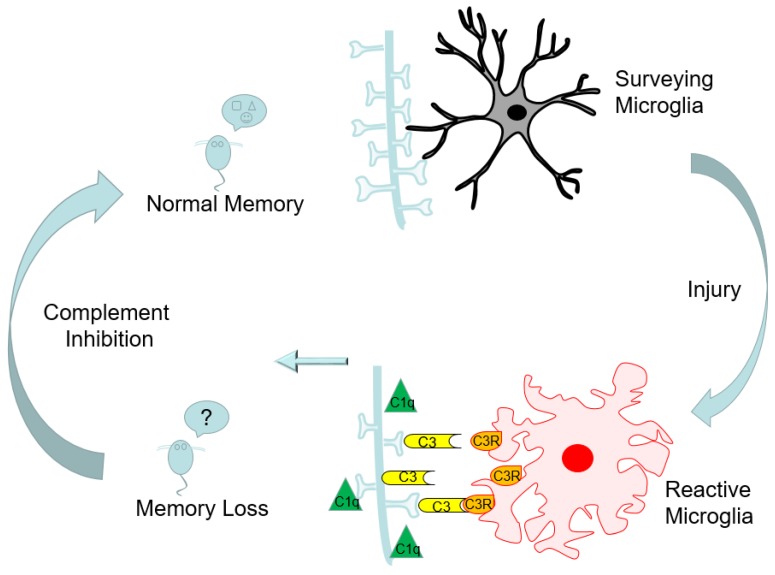
Schematic diagram of the working model. Normal memory function in mice corresponds with numerous synapses and surveying microglia in the hippocampus, the brain region responsible for forming new memories. Traumatic brain injury causes activation of microglia and long-lasting increases in the expressions of C1q and C3 at the synapses in the hippocampus. Microglia expressing the C3R receptor move in and bind the C3-tagged synapses, resulting in phagocytosis of the synapse. The combination of activated microglia and complement-mediated neuronal tagging results in synapse loss in the hippocampus and consequent memory loss. Genetic or pharmacological intervention of the complement pathway prevents trauma-induced memory deficits.

A handful of reports previously addressed the effect of age on the response to traumatic brain injury [7,9,10,28]; however, no studies looked at the chronic long-term consequences of trauma in the aged brain from a mechanistic perspective. In the present studies, we used T_1_- and T_2_-weighted MRI imaging to determine the development of lesion, cavitation, and BBB disruption with time. Importantly, we demonstrated, for the first time, chronic (30 dpi) activation and functional changes in microglial cells, and the critical role that the classical complement cascade plays in the development of chronic memory deficits after injury in aged animals; a schematic representation is shown in Figure 7.

Previous reports using MRI identified lesion formation and BBB breakdown acutely after injury [28,29]; however, limited studies looked at chronic time points after injury. Here, we utilized T_2_-weighted MRI to measure an increase in lesion size and cavitation with time that persisted for up to 28 dpi in the aged animals. Using T_1_-weighted MRI post injection of gadolinium-based contrast agent, we identified that the BBB integrity was chronically compromised, as shown by increased accumulation of contrast agent, indicative of vascular leakage. Overall, we were able to determine that there were long-lasting alterations in lesion size, cavitation, and BBB breakdown at 28 dpi in the aged animals. Previously, it was shown that such changes do not persist in young injured animals [23]. The persistent disruption of protective barrier function may create a permissive environment for exacerbated entry of peripheral immune cells [7] into the brain parenchyma, which contributes to the prolonged inflammatory responses measured here. This information is critical in planning effective therapeutic intervention, suggesting that neuroinflammatory countermeasures may need to be administered for longer periods of time in aged individuals when compared to young individuals.

Previous work from our group and others identified the critical role that peripheral macrophage infiltration into the brain, as well as microglia activation, plays in TBI-related cognitive deficits [7,8,30]. Our recent reports revealed that there is a maladaptive myeloid cell response in the aged injured brain [7,9]; however, these studies focused on acute time points after injury, whereas chronic cognitive deficits are measured several weeks after injury. Importantly, in these earlier studies we demonstrated that age alone modifies the injury-induced inflammatory response as measured by increased macrophage infiltration into the brain when comparing young and old injured animals [7,9]. The exacerbated inflammatory response begins at one dpi and lasts up to a week after injury, further demonstrating a dramatic difference between the immune response in young and old injured animals. Here, we aimed to understand the inflammatory response at a later, chronic time point after injury in the aged brain. Our results demonstrated that there is persistent and significant microglia activation with enhanced phagocytic activity over time (30 dpi). While microglial cell activation occurs rapidly after injury, it is interesting that the heightened engulfment activity was not observed at an early end point (10 dpi). These results indicate that changes in the functionality of the myeloid compartment can progress for a prolonged period of time after injury, and may relate to the fact that aged animals develop worse cognitive deficits after injury compared to young injured animals. In considering therapeutic interventions, these results suggest that a longer or later treatment window might be of benefit when targeting myeloid cell activation.

The complement pathway once thought only to be used by the innate immune system for clearance of pathogens, dying cells, and cellular debris was more recently shown to play a critical role in synapse elimination [13,14,18,19]. Initially identified in development, these findings are now extended to neurodegenerative disorders [15,16,17,20], including Alzheimer’s disease, and recently, a model of nerve injury [31]. Specifically, accumulation of C1q on inactive or weakened synapses leads to removal of these complement tagged of synapses through microglia interactions [13,14,16,17,18,19,31]. Here, we measured increased C1q, C3, and CD11b (with which CD18 forms CR3) expression lasting for up to 30 dpi. Coupled with C1q accumulation on the synapses, we also observed synapse loss and enhanced microglia engulfment capacities in aged injured animals. Taken together, these novel results demonstrate that complement tagging of synapses and increased microglia-engulfing activity are potential regulators of cognitive decline in the aged injured brain.

Age alone was shown to increase complement accumulation (C1q and C3) [18,21], while C1q or C3 deletion in aged animals was shown to prevent glial cell activation and cellular death within the hippocampus. Furthermore, C3^−/−^ knock-out in aged animals preserved cognitive output and increased synaptic puncta and spine density when compared with age-matched controls [18]. The critical role for complement initiation on cognition and synapse numbers was also reported in animal models of Alzheimer’s disease (AD). Deletion of C1q was shown to decrease synapse loss in aged Tg2576 or APP/PS1 mice [32], while C3 deletion was shown to rescue both synapses loss and learning and memory deficits in aged plaque-rich APPswe/PSdE9 transgenic mice [15]. In line with these results, our data suggest that either C3 deletion or C1q blockade protects mice from trauma-induced cognitive decline, possibly through preservation of synapse integrity. However, at this time, we cannot definitively conclude if the restorative effects of complement interference are due to a direct effect on synapse number and function.

Our results demonstrate that TBI-induced complement initiation and increased microglia phagocytic activity in aged animals progress over time, lasting up to 30 days post brain contusion. Importantly, genetic or pharmacological interference of the complement pathway prevents memory decline in aged animals. These novel data suggest that age and injury may have a compounding effect on complement initiation and neuroinflammatory pathways, and propose broader therapeutic options and windows for intervention. In line with our findings, a very recent report demonstrated that blocking formation of the complement membrane attack complex (MAC) reduces acute deficits following severe TBI, whereas inhibition of C3 was required to prevent chronic inflammation and ongoing neuronal loss (at 30 dpi) [33]. These studies, performed in young animals, are consistent with our findings and emphasize the efficacy of blocking the complement cascade. Our study focused on aged animals and demonstrated that specific modulation of the classical complement cascade (via C3^−/−^ or inhibition of C1q) is protective against the loss of cognitive function. C1q is the initiating molecule of the classical complement pathway, and was shown to accumulate on synapses with age, reaching levels up to 300-fold higher than in younger animals [21]. C1q activation leads to tagging of synapses by activated components of C3, which are recognized by the complement receptor CR3 on microglia, leading to synapse elimination.

In conclusion, we demonstrated, for the first time, that (1) TBI-induced long-term memory deficits in aged animals are dependent on accumulation of early complement cascade components (C1q, C3, and CR3) in the brain; (2) there is a progressive increase in synaptic engulfment activity by microglia; and (3) inhibition of the classical complement cascade, either through deletion of C3 or inhibition of C1q, provides protection against cognitive decline (see Figure 7). Because complement activation was previously measured in humans after brain injury [21,34,35], the complement-based interference described here may provide a viable therapeutic option for treatment of TBI in the aging population.

## 4. Materials and Methods

### 4.1. Animals

All experiments were conducted in accordance with the National Institutes of Health Guide for the Care and Use of Laboratory Animals and were approved by the Institutional Animal Care and Use Committee of University of California (San Francisco, CA, USA); animal protocol number 170302, (11 July 2017) and Brigham and Women’s Hospital (Boston, MA, USA, A3431-01), (23 July 2015). Male and female 19-month-old C57B6/J wild-type (WT) mice were obtained from the National Institute of Aging (NIA, Bethesda, MD, USA). Male C3^−/−^ were obtained from Cynthia Lemere, Brigham and Women’s Hospital, Boston, MA, USA.

### 4.2. Trauma Surgery

Aged animals received controlled cortical impact or sham surgeries as previously described [7,23,36].

### 4.3. In Vivo MR Acquisitions

A high-field magnetic resonance system (14.1 tesla, 100 G/cm gradients, Agilent Technologies, Palo Alto, CA, USA) and a proton volume coil (Ø_I_ = 40 mm, Agilent Technologies, Palo Alto, CA, USA) were used to acquire all the MR imaging described in this manuscript.

Anesthesia was performed using isoflurane (1–2% in O_2_). A tail vein catheter (27 Gauge) was first placed to enable subsequent intravenous (iv) injection of Magnevist^®^ for T_1_-weighted MRI, where T1 is the longitudinal relaxation time. Anesthesia was then maintained during all MR acquisitions, while mice were placed in a dedicated cradle inside the MR bore. Physiological monitoring (respiration and temperature) was performed continuously to ensure mice wellbeing and acquisition reproducibilitys.

After initial scout images, high-resolution T_2_ (transverse relaxation time)-weighted anatomical images were acquired using the following parameters: echo time (TE)/repetition time (TR) = 12/2000 ms; slice thickness = 0.5 mm; number of averages = 8; matrix = 256 × 256; field of view (FOV) = 30 × 30 mm^2^; acquisition time = 8 min. Next, BBB lesions were assessed using two identical high-resolution gradient echo sequences separated by 2 min 30 s delay, during which 500 µL of Magnevist^®^ (1 mmol/kg) was injected over 20 s. The corresponding parameters were as follows: TE/TR = 4.6/112 ms; slice thickness = 0.5 mm; number of averages = 30; flip angle = 10; matrix = 256 × 192; field of view (FOV) = 25 × 25 mm^2^; acquisition time = 10 min 41 s.

### 4.4. MR Data Analysis

Using T_2_-weighted images, three regions were manually delineated on each slice and for each animal to create masks for the brain, lesion, and cavitation. The lesion region of interest (ROI) encompasses abnormal looking brain structure around the CCI focal point. Cavitation was defined as hyperintensity replacing tissue around the contusion. All segmentations were performed with ImageJ (University of Wisconsin, Madison, WI, USA). From those masks, three-dimensional (3D) reconstructions were obtained using the AMIRA software (Mercury Computer systems, San Diego, CA, USA).

Using T_1_-weighted images, the enhancement ratio maps were obtained using the following equation:

(1) $$\frac{S_{post}-S_{pre}}{S_{pre}}\times 100$$

where *S_pre_* and *S_post_* represent the signal intensity before and after Magnevist^®^ injection, respectively, at each voxel. Using ImageJ, manual ROIs were drawn around the contusion point (ipsilateral) and reproduced on the opposite side (contralateral). The mean enhancement ratio was computed for each animal in both ROIs.

### 4.5. Tissue Collection

All mice were lethally overdosed and tissues were collected as previously described [27].

### 4.6. qPCR Analysis

Dissected hippocampi from the hemisphere ipsilateral or contralateral to the lesion were used for qPCR analysis as previously described [27].

The primers used were as follows: CD11b, forward (F) 5’–CTGAGACTGGAGGCAACCAT–3’, reverse (R) 5’–GATATCTCCTTCGCGCAGAC–3’; C1qa, F 5’–GTGGCTGAAGATGTCTGCCGAG–3’, R 5’–TTAAAACCTCGGATACCAGTCCG–3’; C3, F 5’–CGCAACGAACAGGTGGAGATCA–3’, R 5’–CTGGAAGTAGCGATTCTTGGCG–3’; glyceraldehyde 3-phosphate dehydrogenase (GAPDH), F 5’–AAATGGTGAAGGTCGGTGTG–3’, R 5’–TGAAGGGGTCGTTGATGG–3’.

### 4.7. Microglia Isolation and Phagocytic Assay

Microglia isolation and flow cytometry analyses were done as previously described with modifications [37]. Briefly, fresh brains were digested into single-cell suspension using the Neural Tissue Dissociation kit (P) (Miltenyi Biotec, Auburn, CA, USA) according to the manufacturer’s instructions. After washing with cold Hanks’ balanced salt solution (HBSS), cells were then resuspended in 30% percoll solution (Sigma-Aldrich, Inc., St. Louis, MO, USA, P4937-100ML) and centrifuged at 800× g for 20 min. Cell pellets were collected, washed, and stained with BV711-CD45 and AF700-CD11b antibodies before analyzing with an Aria III sorter (BD). For the phagocytosis assay, 10 µg of enriched synaptosomes were stained with PSD-95 antibody (1:200, Abcam, Cambridge, UK, ab13552), before being washed, stained with secondary antibody (1:400, goat anti-mouse 488 (Invitrogen, Carlsbad, CA, USA A31556), washed again, and resuspended in 100 µL of sterile phosphate-buffered saline (PBS) before injection. Then, 2 µL of pre-stained synaptosomes were injected into the right hippocampus using the following coordinates, bregma, anteroposterior (AP) −1.6 mm, medialateral (ML) +1.6 mm, and dorsoventral (DV) –2 mm. Mice were euthanized three days post injection and right hemispheres were used for flow cytometry analysis as described above. Data were analyzed with the Flowjo™ software (v10, FlowJo LLC., Ashland, OR, USA). At least 2000 microglia cells (CD45^low^CD11b^+^ events) were collected for the analysis from the dissected ipsilateral hemisphere.

### 4.8. Immunohistochemistry Analysis

For immunohistochemistry analysis, tissue preparation, mounting, and blocking were carried out as previously described [27]. Slides were stained with primary antibodies specific for C1q (Abcam, Cambridge, UK, ab1822451) or PSD-95 (Abcam Cambridge, UK ab13552) overnight, washed three times in Tris-buffered saline (TBS), and stained for the secondary antibody, goat anti-rabbit Alexa-568 (Invitrogen, Carlsbad, CA, USA, A-11011) or goat anti-mouse Alexa-488 (Goat anti-mouse 488 Invitrogen, Carlsbad, CA, USA, A11001). Tissues were fixed using ProLong Gold (Invitrogen, Carlsbad, CA, USA, P36930) and a standard slide cover sealed with nail polish. Two to five images separated by 50–100 µm in the dorsal hippocampus were averaged per animal. For C1q staining, z-stack images were acquired on a Zeiss Imager.Z1 Apotome microscope (Zeiss, Thornwood, NY, USA) controlled by ZEN software (Zeiss 2012, Thornwood, NY, USA) with 200× magnification (C1q). For PSD-95 staining, z-stack images were acquired on a Nikon High-Speed Widefield Confocal microscope (Ti inverted fluorescence; CSU-W1, CSU-W1, Melville, NY, USA) at the UCSF Nikon Imaging Center with 630× magnification.

### 4.9. FLOW Synaptocytometry

Flow synatocytometry was performed as previously described with some modifications [27]. For extracelluar staining, synaptosomes were stained with primary antibodies (C1q-Abcam, Cambridge, UK, ab1822451) for 30 min at 4 °C and agitated after 15 min. Samples were washed twice with 700 µL of antibody staining buffer (PBS + 5% bovine serum albumin (BSA)) at 13,000× *g* for 5 min at 4 °C. Secondary antibodies (Goat anti-rabbit 647, Invitrogen, Carlsbad, CA, USA, A21244) were added for 30 min and agitated after 15 min at 4 °C in the dark. Each day, at least one sham and one TBI sample were analyzed; TBI samples were standardized to the sham group. Samples were run in duplicate. Data were collected on an LSRII (BD) and analyzed with Flowjo™ software (v10, FlowJo LLC., Ashland, OR, USA). Overall, 30,000 events were collected for total synaptosomes, and 50,000 events were collected for C1q-expressing terminals.

### 4.10. C1q Antibody Administration

Beginning immediately after TBI surgery, aged, male animals received intraperitoneal injections of an inhibitory antibody against C1q (ANX-M1, Annexon Biosciences, South San Francisco, CA, USA or an isotype-matched control antibody (mouse immunoglobulin G1 (IgG1), BD Pharmingen, San Diego, CA, USA) [16]. Antibodies were provided by Annexon Biosciences (South San Francisco, CA, USA) and were free of endotoxin and preservatives (stored at 4 °C). Animals received weekly injections (100 mg/kg) until experiment completion (animal termination).

### 4.11. Novel Object Recognition

For all behavioral assays, the experimenters were blinded both to surgery and treatment. One week prior to behavioral analysis, animals were handled for habituation to experimenters and room settings. Behaviors were performed in dark rooms during the animals wake cycle. All behaviors were recorded using an overhead camera connected to a video tracking and analysis system (Ethovision XT 12.0, Noldus Information Technology, Leesburg, VA, USA). When tracking was not optimal, videos were manually scored by an investigator blinded to surgery and treatment.

Recognition memory function was measured beginning at 30 dpi, using a novel object recognition assay (NOR) as previously described [27,38,39].

### 4.12. Data Analysis

Results were analyzed using Prism software (v7.05, GraphPad™; La Jolla, CA, USA) and expressed as mean ± standard error of the mean (SEM). Statistical analyses were performed as listed below with *p* values of <0.05 considered as significant.

## Supplementary Materials

Supplementary materials can be found at http://www.mdpi.com/1422-0067/19/12/3753/s1.
