# Supplementary material for: Traumatic Brain Injury in Aged Mice Induces Chronic Microglia Activation, Synapse Loss, and Complement-Dependent Memory Deficits
Source: Int J Mol Sci. 2018 Nov 26;19(12):3753. doi: 10.3390/ijms19123753 (PMC6321529; doi:10.3390/ijms19123753)
Supplement: Supplementary file 1 [file ijms-19-03753-s001.pdf]

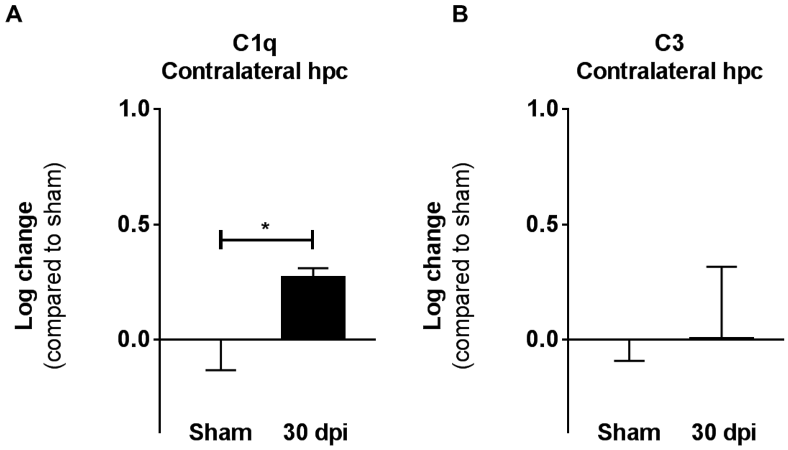

Figure S1. Complement initiation in the hippocampus contralateral to the lesion. (**A**) C1q and (**B**) C3 gene-expression changes in the contralateral hippocampus of aged animals were measured by qPCR analysis, comparing expression levels between sham animals and those 30 days post injury. C1q expression levels increased modestly after injury, and no changes were observed in C3 expression levels. Student's t-test was used to measure differences; $n = 5–10$/group. Bars depict group means and SEM; * $p < 0.05$.
